# Protective mechanisms of nonneutralizing antiviral antibodies

**DOI:** 10.1371/journal.ppat.1011670

**Published:** 2023-10-05

**Authors:** Tawny L. Chandler, Agnes Yang, Claire E. Otero, Sallie R. Permar, Sarah L. Caddy

**Affiliations:** 1 Baker Institute for Animal Health, Cornell University, Ithaca, New York, United States of America; 2 Department of Pediatrics, Weill Cornell Medicine, New York City, New York, United States of America; Mount Sinai School of Medicine, UNITED STATES

## Abstract

Antibodies that can bind to viruses but are unable to block infection in cell culture are known as “nonneutralizing antibodies.” Such antibodies are nearly universally elicited following viral infection and have been characterized in viral infections such as influenza, rotavirus, cytomegalovirus, HIV, and SARS-CoV-2. It has been widely assumed that these nonneutralizing antibodies do not function in a protective way in vivo and therefore are not desirable targets of antiviral interventions; however, increasing evidence now shows this not to be true. Several virus-specific nonneutralizing antibody responses have been correlated with protection in human studies and also shown to significantly reduce virus replication in animal models. The mechanisms by which many of these antibodies function is only now coming to light. While nonneutralizing antibodies cannot prevent viruses entering their host cell, nonneutralizing antibodies work in the extracellular space to recruit effector proteins or cells that can destroy the antibody-virus complex. Other nonneutralizing antibodies exert their effects inside cells, either by blocking the virus life cycle directly or by recruiting the intracellular Fc receptor TRIM21. In this review, we will discuss the multitude of ways in which nonneutralizing antibodies function against a range of viral infections.

## Introduction

An abundance of antibodies are produced following virus infection. The antibodies that can bind viral particles and block entry into cells are known as “neutralizing antibodies.” The antibodies that can bind viral particles but do not prevent infection in vitro are called “nonneutralizing antibodies” (nNAbs). It is often assumed that only neutralizing antibodies are important in mediating protection against viral infection; however, it is now increasingly clear that nNAbs can also play a key role in protecting hosts from viral infection.

Production of nNAbs has been recognised in response to virus infection for decades. While their relevance has often been overlooked, as early as 1982, it was experimentally demonstrated that a monoclonal nNAb against E1 glycoprotein of Sindbis virus was protective in mice [1]. Since then, a substantial number of studies have undoubtedly proven that monoclonal nNAbs against diverse viruses can prevent disease in a range of animal models (Table 1).

**Table 1:** Viruses experimentally proven to be protected against by monoclonal nNAbs. The studies included were all performed by passive transfer of antibody and virus challenge in animal models, and each represents the first report for each virus type.

Virus		Viral nNAb target	Protective mechanism in vivo	Year	Reference
**negative ssRNA**	Influenza	Nucleoprotein	Not determined	2008	[7]
	Lymphocytic choriomeningitis virus	Nucleoprotein	Not determined	2013	[8,9]
	Crimean-Congo hemorrhagic fever virus	Glycoprotein 38	Complement-mediated	2019	[10]
	Ebola virus	Glycoprotein	Not determined	2000	[11]
	Marburg	Glycoprotein 2	FcγR-mediated	2020	[12]
	Sendai virus	F protein	Not determined	1990	[13]
**postive ssRNA**	Coxsackie virus	VP2 capsid protein	Fc-mediated	2022	[14]
	SARS-CoV-2	Nucleoprotein	Not determined	2022	[15]
	Murine hepatitis virus	E2 glycoprotein and nucleoprotein	Not determined	1986	[16]
	Sindbis virus	E1 glycoprotein	Not determined	1982	[1]
	Semliki Forest virus	E2 glycoprotein	Not determined	1983	[17]
	Mayaro virus	E2 glycoprotein	FcγR-mediated	2021	[18]
	West Nile virus	NS1 protein	FcγR-mediated	2006	[19]
	Yellow fever virus	Envelope and NS proteins	Not determined	1986	[20]
	Zika virus	NS1 protein	FcγR-mediated	2018	[21]
	Lactate dehydrogenase-elevating virus	VP3 env glycoprotein	Not determined	1987	[22]
**dsRNA**	Rotavirus	VP6 inner capsid protein	Intracellular	1996	[23]
**dsDNA**	Herpes simplex virus 2	Glycoproteins A, B, C, D, E, F	Not determined	1982	[24]
	Murine cytomegalovirus	Glycoprotein B	Not determined	2017	[25]

dsDNA, double stranded DNA; dsRNA, double-stranded RNA; nNAb, nonneutralizing antibody; SARS-CoV-2, Severe Acute Respiratory Syndrome Coronavirus 2; ssRNA, single-stranded RNA.

NNAbs have now been correlated with protection from viral infection and/or disease in several human studies. Clinical trials for vaccines targeting influenza virus [2] and HIV [3] found that protection was associated with nNAbs. Similarly, partial vaccine-mediated protection against human cytomegalovirus (HCMV) [4] and reduced risk of in utero HCMV transmission [5,6] has been associated with nNAbs.

## Mechanisms of nNAb-mediated protection

**Fig 1:**
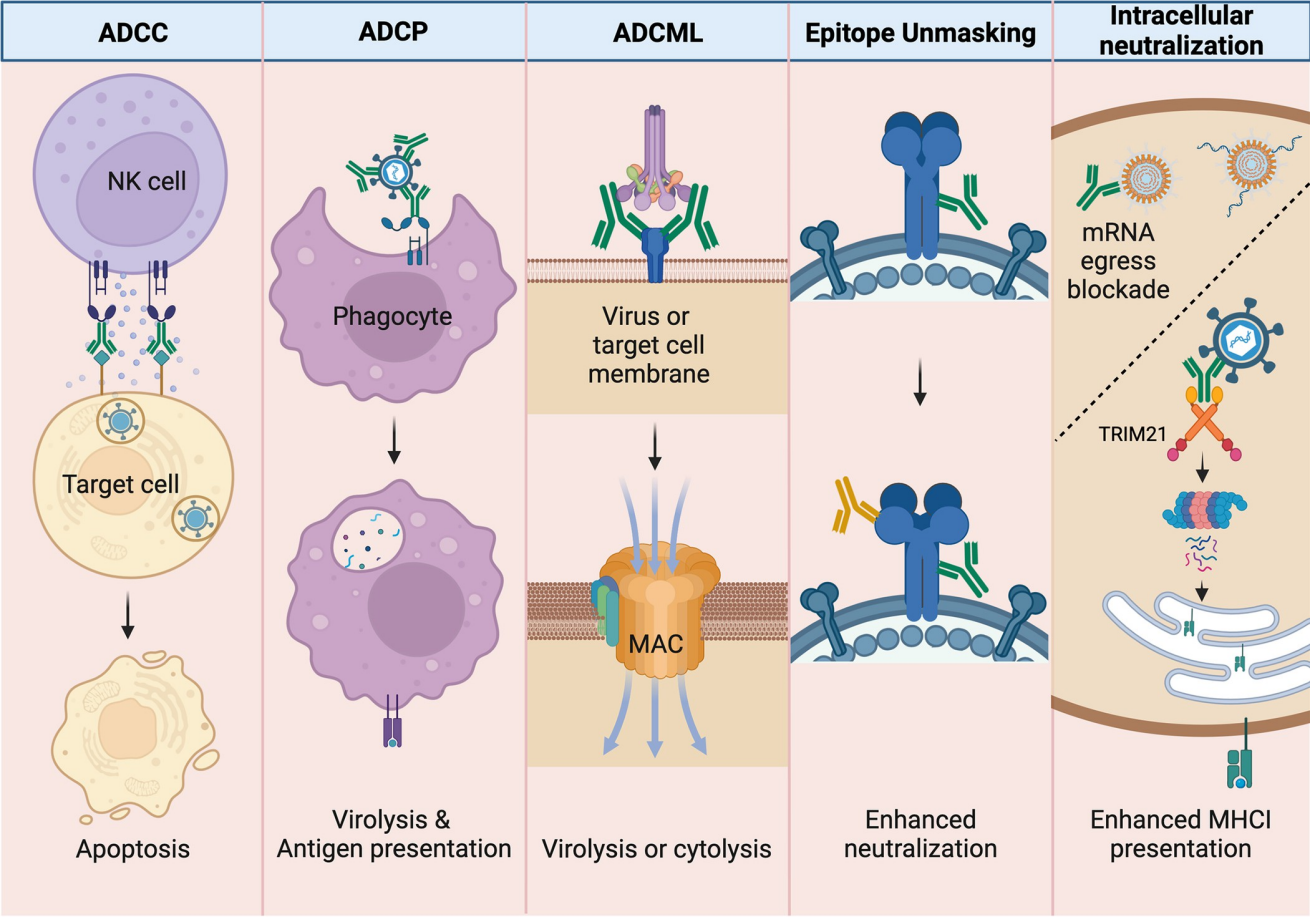
nNAb-mediated antiviral effector functions following antigen binding to Fab. NK cells can exhibit **ADCC** by detecting target cells (i.e., virus-infected cells) opsonized by antibodies via the FcγRIII (CD16) receptor and induce apoptosis by releasing cytotoxic granules. Macrophages and other phagocytes perform **ADCP** by recognizing opsonized viral particles via the FcγRI (CD64) and FcγRIIA (CD32) receptors leading to virolysis and downstream antigen presentation of viral antigen. Antibodies activate the classical complement pathway after binding to the soluble complement complex, C1q. In addition to viral aggregation and opsonization, antibody-dependent complement fixation on viral or target cell membranes can lead to the formation of the pore-forming MAC and **ADCML**. Cooperation between nNAb (green) binding that exposes epitopes for neutralizing antibody (yellow) binding can enhance the efficacy of virus neutralization. Antibodies can block viral replication intracellularly; for dsRNA viruses that maintain an intact innermost capsid inside cells, antibodies can block mRNA egress. Intracellular antibodies can also be bound by TRIM21, which leads to proteasomal degradation of the virus–antibody complex and can result in enhanced MHC class I antigen presentation. Created with Biorender.com. ADCC, antibody-dependent cellular cytotoxicity; ADCML, antibody-dependent complement-mediated lysis; ADCP, antibody-dependent cellular phagocytosis; MAC, membrane attack complex; NK, natural killer; nNAb, nonneutralizing antibody.

It is now clear that nNAbs function via a variety of mechanisms (Fig 1). Some mechanisms rely on extracellular effector cells or proteins, whereas others are mediated by intracellular activity. The majority of these functions require engagement of the constant (“Fc”) region of antibodies. For some nNAbs, the exact mechanisms of action are still unclear, whereas others function via multiple Fc-mediated mechanisms. It is important to note that many of the Fc-mediated effector functions of nNAbs can also be mediated by neutralizing antibodies and are not necessarily exclusive to nNAbs. In fact, optimal protection by neutralizing antibodies against influenza [26] and HIV [27] infections is evident when antibodies also engage Fcγ receptors.

### Antibody-dependent cellular cytotoxicity (ADCC)

ADCC is canonically mediated by FcγRIII (CD16) expressed on granulocytes, such as natural killer (NK) cells and neutrophils. Engagement of CD16 by immunoglobulin G (IgG) complexed to an infected cell promotes the release of cytotoxic granules, a highly regulated process that induces apoptosis in the infected cell [28]. Assays measuring ADCC evaluate surrogate markers that quantify target cell viability or apoptosis, or effector cell degranulation through CD107a expression or granzyme release [29,30].

Fc-mediated effector responses, and ADCC in particular, have been thought to play a major role in control of infections that utilize cell-to-cell spread as a primary mode of dissemination, such as herpes simplex virus (HSV) and cytomegalovirus (CMV). Both of these viruses can cause severe disease in infected neonates, but maternal antiviral ADCC-mediating antibodies have been associated with protection from disseminated HSV infection and from vertical CMV transmission in clinical observational studies [6,31].

Antibodies capable of mediating ADCC have also been shown to be elicited by many RNA viral infections, including influenza, HIV, Severe Acute Respiratory Syndrome Coronavirus 2 (SARS-CoV-2), hepatitis C virus, and respiratory syncytial virus (RSV) [32–36]. In animal models, SARS-CoV-2 nucleoprotein-targeting nNAbs that induced ADCC in vitro were shown to reduce viral loads when passively transferred to mice prior to virus challenge [15]. This is likely due to surface expression of nucleoprotein [37], and indeed, nNAbs targeting NS1 of Flaviviruses, which is also detectable on the cell surface, have demonstrated protection in vivo [19,21].

### Antibody-dependent cellular phagocytosis (ADCP)

ADCP is mediated by engagement of IgG immune complexes with FcγRI (CD64) and FcγRIIA (CD32A) expressed on phagocytic effector cells, such as monocytes, macrophages, and dendritic cells [38]. Through engulfment and fusion with lysosomes to destroy immune complexes, phagocytosis reduces the amount of infectious material. Further, macrophages and dendritic cells are involved in antigen presentation of peptides from degraded pathogens on major histocompatibility complex molecules to T cells [38]. ADCP assays measure uptake of opsonized particles, such as whole virions or beads coated with specific antigens of interest, or removal of infected target cells [39,40].

ADCP has been associated with protection from vertical CMV transmission in a human observational study [5] and additionally implicated in reducing the risk of CMV viremia in lung transplant recipients [41]. ADCP has also stood out as associated with reduced disease risk in HIV vaccine trials, which has been corroborated by studies in the rhesus macaque model [42]. In addition, greater ADCP function was associated with reduced mortality from SARS-CoV-2 infection [43].

As both ADCP and ADCC require FcγR interactions, many studies do not easily differentiate between the two and can only conclude that FcγR effector functions are required for nNAb-mediated protection. An exception to this was a comprehensive analysis of influenza-specific monoclonal nNAbs derived from vaccinated humans; nNAbs induced robust phagocytosis, but not ADCC in vitro, and were protective in mice challenged with H7N9 influenza [44].

### Complement activation

Antibody binding to viral epitopes can activate the classical complement pathway via C1q. Binding of C1q to antigen–antibody complexes leads to formation of C3 convertase and deposition of C3b on membrane surfaces. This can result in virus elimination by opsonization and phagocytosis, or target cells and enveloped viruses may undergo osmotic lysis due to formation of the membrane attack complex (MAC) [45].

As complement activation requires a source of complement proteins, cell-based neutralization assays do not capture antibody-dependent complement-mediated virolysis or cytotoxicity that may be beneficial in vivo. When complement was added to a standard neutralization assay, monoclonal nNAbs to Sendai and Ebola Zaire virus demonstrated complement-mediated virolysis and robust neutralization in vitro. These nNAbs were subsequently shown to be protective following passive transfer in murine models [11,13]. Studies with a monoclonal nNAb against West Nile virus envelope protein also showed complement-dependent and Fcγ receptor–dependent protection in mice [46].

Some nNAbs that confer protection with complement activation have been shown to be cross-reactive and cross-protective across virus genera. For example, passively transferred nNAbs that activated complement and conferred protection against Sindbis virus challenge in a murine model were also protective against lethal western equine encephalitis challenge in mice [1], and passive transfer of IgG purified from dengue virus–immune hamsters were poorly neutralizing but cross-reactive and protective against West Nile virus in mice [46].

### Epitope unmasking

Several studies have identified an unexpected synergy between neutralizing and nNAbs. Howell and colleagues demonstrated that nNAbs improved the efficacy of neutralizing antibodies against Ebola and Sudan virus in vitro [47] and extended these findings in vivo, demonstrating cooperative protection by a neutralizing and nNAb pair in Ebola virus–infected mice. It was consequently suggested that nNAbs may improve the efficacy of neutralizing antibodies by altering or exposing viral epitopes.

Epitope unmasking by a nNAb was subsequently experimentally proven using a neutralizing/nNAb combination specific for Marburg virus glycoprotein. The nNAb increased the accessibility of neutralizing antibody epitopes in the receptor-binding site to enhance neutralization [12]. Synergy between a nNab and a neutralizing antibody has also been identified in SARS-CoV-2–infected mice. A nNAb mutated for enhanced FcγR binding and ADCC moderately decreased SARS-CoV-2 spread but conveyed complete protection when combined with a nonprotective neutralizing MAb lacking any Fc-effector function [48]. Although exact mechanisms of action were undetermined, it is possible that alteration of neutralizing epitopes were contributing to this protection.

### Intracellular neutralization

Antibodies can enter cells via a number of routes including transcytosis and by attachment to viruses [49]. Thus far, nNabs have been shown to disrupt viral replication inside cells via one for double-stranded RNA (dsRNA) viruses, and another for an enveloped virus.

MAbs targeting the inner capsid protein (VP6) of a dsRNA virus in the genus *Rotavirus* were first shown to be nonneutralizing yet protective in a seminal paper from the Greenberg lab. Subsequent work found that protective anti-VP6 antibodies block pores in the inner capsid, which is only exposed inside cells [50]. The replication cycle of dsRNA viruses is unusual in that transcription occurs within the intact inner capsid in the cytoplasm, and newly transcribed mRNA must egress through capsid pores. Pore blockade by antibodies will therefore prevent mRNA escape. This mechanism represents a rare example of a nNAb where the variable region plays a critical role.

A function for the Fc region of VP6-specific nNAbs was discovered more recently. It was demonstrated that a VP6-specific nNAb could activate the unique intracellular antibody receptor TRIM21 [51]. TRIM21 is an E3 receptor ligase that can bind to the Fc portion of antibodies through its PRYSPRY domain [52]. Binding induces auto-ubiquitination and recruitment of the proteasome to the virus–antibody complex, resulting in virus degradation.

nNAbs targeting viral nucleoproteins (also known as nucleocapsid, NP, or N-protein) are commonly induced following infection with several enveloped viral families as shown in Table 1. Antibody-mediated neutralization cannot occur in vitro as the viral capsid shields the nucleoprotein in intact particles. Nevertheless, there are several studies that demonstrate nucleoprotein-specific nNAbs are protective in vivo [7–9,15,16].

ADCC, ADCP, and complement activation have been shown to be irrelevant for the protective effect of nNAbs specific for lymphocytic choriomeningitis virus (LCMV) [8,9]. Instead, a novel role for TRIM21 was identified, whereby nNAb-mediated protection was lost in TRIM21-knockout mice [53]. Furthermore, nucleoprotein-specific antibodies were positively associated with enhanced nucleoprotein-specific CD8+ T cell activation in the presence of TRIM21. It was proposed that TRIM21-mediated degradation was leading to enhanced presentation of nucleoprotein-specific peptides on MHC class I molecules. Independent identification of a key role for macrophages in nNAb-driven protection against LCMV supports a role for antigen-presenting cells in this pathway [54].

Nucleoprotein-specific nNAbs from viruses other than LCMV have also been postulated to work in a similar TRIM21-dependent manner [49]. Studies with influenza virus have identified nucleoprotein-specific nNAbs that are protective against heterologous virus strains [7], and an association between CD8+ T cell activation, macrophages, and antigen presentation has been demonstrated [55].

## Conclusions

Antibodies can mediate protection against viruses via a wide array of strategies not captured by classical neutralization assays. This has historically led nNAbs to be overlooked, but it is now evident than some nNAbs can be highly protective. Whereas the rudimentary principles of some nNAb functions have been apparent for decades, significant advances in mechanistic understanding have been made in recent years. This has been supported by development of many new high-throughput nonneutralizing assays [56].

Greater understanding of nNAb activity is important when determining correlates of protection from infection and vaccination. It is now clear that measurement of nNAb activity should be taken into consideration in all vaccine clinical trials. Attention to development of vaccine strategies that can boost nNAb activity may also prove valuable for management of viral infections. The nonneutralizing functions of MAb therapeutics are similarly now becoming more widely appreciated. Therapies that combine neutralizing and nonneutralizing MAbs have been developed, and this strategy has documented protection against Ebola virus in nonhuman primates [57]. In addition, engineering therapeutic MAbs to have enhanced nonneutralizing functions is now more commonplace, leading to increased overall antibody potency [58].

Improved awareness of the significance of nNAbs is going to require a multifaceted approach; ongoing research into new mechanisms, continued development of nonstandard functional assays, and extensive testing of clinical samples following virus infection or vaccination. Together, these will be essential to improve our knowledge and leverage these antiviral antibodies for improved vaccines and immunotherapies.
